# The Tragedy Caused by Fake Antimalarial Drugs

**DOI:** 10.4084/MJHID.2012.027

**Published:** 2012-05-04

**Authors:** Pierre Ambroise-Thomas

**Affiliations:** National French Academy of Medicine

## Abstract

Counterfeit antimalarials (mainly artemisinin derivatives) is a crucial health problem in developing countries, particularly in Africa. The illegal production, sale and distribution of fake drugs is a huge market evaluated to several billion of dollars and represents more than 50% of the pharmaceutical market in several African countries. Fake drugs have led to a very great number of deaths from untreated malaria or fatality provoked by toxic ingredients. These fake medicines increase the risk of artemisinin resistance developed by the use of sub therapeutic dosages of antimalarials. Tackling this criminal traffic is the objective of an international program created by WHO and involves the international police and custom organizations like INTERPOL. Several very important and encouraging results have been obtained, but the problem will be completely solved if genuine antimalarials, free-of-charge, are handed-over to populations in sub Sahara African countries.

For decades, counterfeiting of drugs was almost ignored or at least largely underestimated. It has now clearly emerged as one of the most crucial public health problems mainly for malaria treatment in developing countries.^1^,^2^,^4^,^7^,^8^ The counterfeit of antimalarial drugs at first received little attention compared with considerable efforts made in other aspects of malaria control. Although important efforts have been made to fight against this criminal traffic, much more needs to be done.

It is interesting to note that during the past centuries, poor quality drugs have been a persistent problem starting in the 1600s with fake cinchona bark and in the 1800s with fake quinine. The problem of modern-day counterfeit drugs was first addressed in 1985 at a conference in Nairobi. Unfortunately, the counterfeit market continued to grow and has considerably spread all over the world during the last decade, particularly in developing countries very often with the most dramatic consequences. This is largely the result of the wide-spread use of the internet to market counterfeit products in an unregulated environment of anonymity. The World Health Organization (WHO) estimates that 50% of medicines available via internet are fake.

On the whole, counterfeiting of drugs is a huge market estimated by WHO at more than US$35 billion and represents more than 15% of the pharmaceutical market worldwide with this proportion rising to more than 60% in developing countries (35–90% in south East Asia).^11^ Pharmaceutical products are attractive candidates for producers involved in this illegal trade mainly because the benefits are immense and the risks very limited as they almost always prepare large batches of fake drugs before disappearing and going elsewhere to start-up the same traffic all over again. Fake drugs are easily transportable, have high value per unit, and above all, their quality cannot be scientifically or legally assessed without a quality testing laboratory.

Almost all kinds of medicines are counterfeited, but antimalarials and particularly artemisinin derivatives are more vulnerable to counterfeiting. Until now artemisinin is mainly (if not only) produced in Asia with unfortunately a very large proportion of fake products. According to an account of the Operation Jupiter programme published in the open-access journal *PLoS Medicine,* 30 to 35% of bought artesunate are counterfeit in South East Asia (Thai-Myanmar border, Laos, Cambodia and Vietnam).^11^ A report was presented by Interpol to the Chinese Ministry of Health which launched a criminal investigation resulting in the arrest of a native of Yunnan province alleged to have bought 240 000 blisters of counterfeit artesunate.

But the situation is even more dramatic in sub Saharan Africa where the burden of malaria is the greatest. In 2001, WHO recommended that malaria-endemic African countries consider changing to artemisinin-derivatives, based of combination therapy (ACT) as first line treatment for malaria. This change has gradually been implemented (correct as recast?) during the past years, but adopting this new policy was not easy because of the high cost of ACT and the relative shortage of the raw plant material (*Artemisia annua*) from which artemisinin is extracted. Probably more than 200 million courses of ACT are annually used in Africa and the high cost and shortage of ACT provide favorable platforms for the spread of fake artemisinin that can put the lives of many thousands African young children at risk. A large proportion of these fake antimalarials which are genuinely fake are imported from Asia. There is also a thriving fake antimalarial drug industry in some African countries, where governments promote locally produced antimalarials which are not always counterfeited, *stricto sensu*, but sub-standard or fake drugs, with a name, label, packaging different from that of the genuine products.

Counterfeiting is more than a criminal act. *Manslaughter*^11^ is perfectly justified to describe such an act although some prefer calling it simply *murder*. It always involves unscrupulous people directing a highly organized technical and sophisticated criminal trade. These criminals make tablets out of starch, chalk, and a variety of wrong active ingredients for a life-threatening disease that particularly affects the poor populations who can least afford. These perpetrators knowingly manufacture these fakes medicines and they fully know that their ineffective product may kill people who would otherwise survive their malaria infection.

In counterfeit products, they are no or sub-therapeutic quantities of artemisinin derivatives and sometimes contain potentially dangerous substances such as metamizole (which may lead to bone marrow failure with agranulocytosis), melamine (which causes very severe and sometimes lethal urinary lithiasis) or safrole, whose hepatocarcinogenetic action was detected in 1960–1961, after it had been used as a flavoring agent for beverages

The full extent of this deceptive yet lucrative criminal activity is not well known. Figures are underestimated and the consequences of this criminal activity certainly go beyond human boundaries.^8^ Fake drugs have led to a very great number of deaths (unfortunately impossible to know how many even by estimates or even to evaluate) from untreated malaria or provoked by toxic ingredients as these fake medicines increase the risk of artemisinin resistance as a result of the use of sub therapeutic dosages of antimalarials.

But some other important consequences of the artemisinin counterfeiting are more indirect and often ignored or underestimated. Fake products cause the multiplication of false reports on artemisinin resistance and deplete the public's confidence in this vital drug and in the pharmaceutical industry.^9^ On the other hand, large economic losses for legitimate manufacturers demotivates the pharmaceutical industry in producing tropical medicines, which is already by far not a top priority for this industry.

The traffic of fake antimalarials is evidently linked to the dramatic widespread prevalence of malaria in many countries and the need for an affordable and active treatment. But officials of non-governmental organizations (NGO’s), international agencies or aid organizations, through support of the Global Fund have initiated some incentives. They want to increase competition and lower the prices of medicines but they rarely take note of the counterfeit market these incentives can encourage. After an active information campaign during the past years, some of the associations and agencies are more careful; but the problem still exists with small NGOs who have scant experience of the health and economic realities in developing countries.

To tackle the trade in counterfeit drugs it is ideal to entirely dismantle this criminal market and to know the true scale of the issue. In recent years, increasing numbers of substandard and fake medications were detected in the international markets, but precise figures of the global situation are lacking, particularly in rural areas of Africa. Indeed, identical holograms, batch numbers and expiry dates, and blisters and tablets looking absolutely genuine, make detection of fake drugs particularly difficult. Their packaging is always a perfect copy and their quality cannot be assessed readily by lay persons or even experts of pharmaceutical industry without the aid of a quality testing laboratory. To check the product(s) inside the blisters, many techniques were proposed, the most precise being of course the most sophisticated (for example, the innovative high-performance liquid chromatography method, two-dimensional diffusion-ordered nuclear magnetic resonance spectroscopy, combined to imaging desorption electrospray ionization mass spectrometry, and direct analysis in real-time mass spectrometry).^6^,^15^,^16^ These obviously require laboratories with high level of technical facilities which are rarely available in developing countries.

But like seeing the ears of the hippopotamus, fake drugs detection is only the first step. In almost all cases, it is after all impossible to identify the manufacturers except in exceptional situations as was the case in China, where a rare pollen specific in an area was detected by palynology^18^ in fake artemisinins and allowed the authorities to arrest those involved in this criminal activity. Very often even when the brain behind this lucrative business is identified, it is generally impossible to arrest him before he slips away.

The only practical possibility is to act at the wholesale level and intercept and destroy fake drugs. Last year, 45 countries took part in an internet-based campaign (operation PANGEA III) involving several international organizations, including police, customs, national drug regulators and internet service providers. These recent global enforcement efforts have led to the arrest of online counterfeit sellers. However this action did not stem out supplies from illegal online sellers or keep them away from their creativity in illegally selling their products.

Although the internet plays a major role in this traffic, it is not the only mean used by wholesalers. To tackle drugs counterfeiting other solutions have to be adopted in developing countries, mainly in Africa, at the multinational or national level. Some notable success were obtained, for example in Nigeria by National Agency for Food, Drug Administration and control (NAFDAC) or mainly thanks to an international organization, IMPACT (International Medical Products Anti Counterfeiting Task Force) created by WHO in 2008, closely with INTERPOL and involving a total of 193 countries. In East Africa, a sub program of IMPACT, (operation MAMBA)^13^,^14^ led to the to arrest several manufacturers, wholesalers and retail pharmacists. It also allowed the seizure of destruction of counterfeit drugs valued at several billion of dollars. But at the end of the distribution chain, it is difficult and almost impossible to obtain significant results in Africa, where counterfeit drugs are often sold alongside vegetables in local markets or by hawkers (sidewalk markets)

Tackling this network of criminal organizations at different levels is essential but not enough as it requires other approaches different from law enforcement and monitoring. One such measure could involve increasing the availability of low priced genuine drugs –particularly antimalarials - in developing countries. However, the risk is even with low prices, it will be easy for counterfeiters to produce and sell fake drugs at a much cheaper rate, because their main (if not only) expense is packaging.^10^

Finally, the solution would be to give antimalarials completely free of charge to developing countries which may imply a huge and perhaps unrealistic financial effort.

## References

[1] Aldhous P (2005). Murder by Medicines, News Feature. Nature.

[2] Ambroise-Thomas P (2010). Contrefaçons, malfaçons : les faux médicaments qui tuent. Méd.Trop.

[3] Amin Aa, Kokwaro GO (2007). Antimalarial drug quality in Africa. J Clin Pharm Ther.

[4] Bate R, Coticelli P, Tren R, Attaran A (2008). Antimalarial drug quality in the most severely malarious parts of Africa - a six country study. PLoS ONE 2008.

[5] Bate R, Attaran A (2010). A counterfeit drug treaty: great idea, wrong implementation. Lancet.

[6] Debrus B, Lebrun P, Kindenge JM, Lecomte F, Ceccat A, Caliaro G, Mbay JM, Boulanger B, Marini RD, Rozet E, Hubert P (2011). Innovative high-performance liquid chromatography method development for the screening of 19 antimalarial drugs based on a generic approach, using design of experiments, independent component analysis and design space. J Chromatogr.

[7] Fernandez Fm, Hostetler D, Powell K, Kaur H, Green MD, Mildenhall DC, Newton PN (2011). Poor quality drugs: grand challenges in high throughput detection, countrywide sampling, and forensics in developing countries. Analist.

[8] Mackey TK, Lian BA (2011). The global counterfeit drug trade: patient safety and public health risks. JPharmSci.

[9] Nair A, Strauch S, Jahnka RW, Dressman J (2011). Are counterfeit or substandard anti-infective products the cause of treatment failure in Papa New Guinea?. J.Pharm.Sci.

[10] Newton PN, Green MD, Fernandez FM, Day JP, White NJ (2006). Counterfeit anti-infective medicines. Lancet Infect Dis.

[11] Newton PN, Mcgready R, Fernandez F, Green MD, Sunjio M (2006). Manslaughter by Fake Artesunate in Asia—Will Africa Be Next?. PLoS Med.

[12] Nyadong L, Harris GA, Balayssac S, Galhena AS, Malet-Martino M, Martino R, Parry Rm, Wang Md, Fernandez Fm, Gilard V (2009). Combining two-dimensional diffusion-ordered nuclear magnetic resonance spectroscopy, imaging desorption electrospray ionization mass spectrometry, and direct analysis in real-time mass spectrometry for the integral investigation of counterfeit pharmaceuticals. Anal Chem.

[13] Operation Jupiter rains on fake antimalarials (2008). A multinational, multidisciplinary initiative involving scientists, public health workers, police and government officials could provide a cogent model for rooting out and stamping out counterfeiting of essential and lifesaving drugs. http://www.in-pharmatechnologist.com.

[14] Operation Mamba (IMPACT) – targeting counterfeit medicines in Tanzania and Uganda. http://www.interpol.int/public/news/2008/mamba20081029.asp.

[15] Ricci C, Nyading L, Yang F, Fernandez FM, Brown CD, Newton PN, Kazarian SG (2008). Assessment of hand-held Raman instrumentation for in situ screening for potentially counterfeit artesunate antimalarial tablets by FT-Raman spectroscopy and direct ionization mass spectrometry. Anal Chim Acta.

[16] Ricci C, Eliasson C, Mcleod NA, Newton PN, Matousek P, Kazarian SG (2007). Characterization of genuine and fake artesunate anti-malarial tablets using Fourier transform infrared imaging and spatially offset Raman spectroscopy through blister packs. Anal Bioanal Chem.

[17] Siva N (2010). Tackling the booming trade in counterfeit drugs. Lancet.

[18] Stanley EA (1992). Application of palynology to establish the provenance and travel history of illicit drugs. Microscope.

